# Association of Melatonin Pathway Gene's Single-Nucleotide Polymorphisms with Systemic Lupus Erythematosus in a Chinese Population

**DOI:** 10.1155/2019/2397698

**Published:** 2019-11-13

**Authors:** Peng Wang, Lei Liu, Li-Fang Zhao, Chan-Na Zhao, Yan-Mei Mao, Yi-Lin Dan, Qian Wu, Xiao-Mei Li, De-Guang Wang, Hai-Feng Pan

**Affiliations:** ^1^Center for Genetic Epidemiology and Genomics, School of Public Health, Medical College of Soochow University, 199 Renai Road, Suzhou, Jiangsu, China; ^2^Department of Epidemiology and Biostatistics, School of Public Health, Anhui Medical University, 81 Meishan Road, Hefei, Anhui, China; ^3^Department of Parasitology, Medical College of Soochow University, 199 Renai Road, Suzhou, Jiangsu, China; ^4^Anhui Province Key Laboratory of Major Autoimmune Diseases, 81 Meishan Road, Hefei, Anhui, China; ^5^Department of Rheumatology and Immunology, Anhui Provincial Hospital, 17 Lujiang Road, Hefei, Anhui, China; ^6^Department of Nephrology, The Second Affiliated Hospital of Anhui Medical University, Hefei, Anhui, China

## Abstract

**Objectives:**

This study was to investigate the association of melatonin (MTN) pathway gene's single-nucleotide polymorphisms (SNPs) with susceptibility to systemic lupus erythematosus (SLE).

**Methods:**

We recruited 495 SLE patients and 493 healthy controls, 11 tag SNPs in *MTN receptor 1a* (*MTNR1a*), *MTNR1b*, and arylalkylamine N-acetyltransferase (*AANAT*) genes were genotyped and analyzed. Serum MTN concentration was determined by enzyme-linked immunosorbent assay (ELISA) kits.

**Results:**

Two SNPs of *AANAT* gene (rs8150 and rs3760138) associated with the risk of SLE; CC carriers of rs8150 had a lower risk as compared to GG (OR = 0.537, 95% CI: 0.361, 0.799), whereas GG carrier in rs3760138 had an increased risk (OR = 1.823, 95% CI: 1.154, 2.880) compared to TT. However, we did not find any genetic association between the other nine SNPs with SLE risk. Case-only analysis showed associations of rs2165667 and rs1562444 with arthritis, rs10830962 with malar rash, rs3760138 with immunological abnormality, and rs8150 with hematological abnormality. Furthermore, a significant difference between plasma MTN levels with different genotypes of rs1562444 was observed. Haplotype analyses revealed that haplotype of CCTAT, CTAGT, and GGG was significantly associated with the increased risk in SLE susceptibility, but TCTAT and CTG appeared to be a protective haplotype.

**Conclusions:**

The present study supported the genetic association of MTN pathway genes with SLE susceptibility and specific clinical manifestations, suggesting the potential role of MTN pathway genes in the pathogenesis and development of SLE.

## 1. Introduction

Systemic lupus erythematosus (SLE) is a chronic and inflammatory autoimmune disease characterized by antinuclear autoantibody production and a multitude of immune-complex deposition, which is involved in multisystem; such as the skin, kidney, and brain; and caused organs/tissue destruction [1, 2]. Until now, the etiology of SLE is still not fully understood. A large body of literatures have suggested that the interactions between exogenous (infectious triggers, ultraviolet radiation, and dietary habit) and endogenous sources (hereditary susceptibility, endocrine disorders and disturbed status of oxidative metabolism, and autoimmune responsiveness sympathetic nervous system) are responsible for the pathogenesis and development of SLE [3–9].

Melatonin (MTN), as one of the major neuroendocrine hormones, is mainly produced and secreted by the pineal gland; it allows to regulate the circadian day-night rhythm and seasonal biorhythms and is also a key player in the neuroendocrine-immune pathway [10, 11]. Several studies have suggested the important role of MTN on the regulation of the immune system; it can skew the immune response by repressing the production of several proinflammatory cytokines (such as tumor necrosis factor- (TNF-) *α*, interleukin- (IL-) 1*β*, and IL-6), as well as blocking the DNA-binding activity of NF-*κ*B both *in vitro* and *in vivo*, exerting an anti-inflammatory effect [11–16].

Previous studies have investigated the underlying role of MTN in SLE. Lechner et al. observed an increased MTN level in MRL/MP-fas lupus-prone mice (represents an animal model for human autoimmune diseases, which spontaneously develops lupus-like glomerulonephritis, systemic vasculitis, arthritis, and sialadenitis) during the light phase; under the regulation of MTN, the levels of autoantibodies were reduced, and the histological changes were improved in female lupus MRL/MP-fas lupus-prone mice [17]. MTN is able to inhibit IgM, anti-dsDNA, and anti-histone antibodies, thus, decreases the levels of IL-6 and IL-13 and increases the IL-12 levels [18]. In patients with SLE, a lower daily MTN level was observed as compared to healthy controls, and this decreased daily MTN level inversely correlated with the systemic lupus erythematosus disease activity index (SLEDAI) [19]. In addition, study has also revealed the seasonal pattern of MTN levels in SLE, with an elevated daily plasma MTN levels in December than in June [20]. These findings provided the possible evidence that MTN might play a potential role in the pathogenesis of SLE.

The synthesis and function of MTN mainly depend on three MTN pathway genes, *MTN receptors 1a/1b* (*MTNR1a/MTNR1b*) are largely responsible for mediating the downstream effects of MTN, and arylalkylamine N-acetyltransferase (*AANAT*) is the major enzyme in MTN synthesis [21, 22]. The genetic association of MTN genes with some diseases has been demonstrated in several studies, including SLE, multiple sclerosis (MS), breast cancer, and major depression [23–26]. Nevertheless, associations between genetic variation in MTN pathway genes and SLE susceptibility have not been determined.

In the present study, we conducted a case–control study to comprehensively evaluate the role of common genetic variation in the *MTNR1a*, *MTNR1b*, and *AANAT* genes to SLE susceptibility in a Chinese population.

## 2. Materials and Methods

### 2.1. Study Subjects and Methods

This case–control genotyping study recruited a total of 988 subjects (495 SLE patients and 493 healthy controls). The sample size and power calculation of the study were computed by power and sample size program, where both the minor allele frequency (MAF) and statistical significant level were set as 0.05, odds ratio (OR) was 1.5, case and control ratio was 1 : 1 and when the statistical power was 0.8, the computed sample size for cases was 451. In addition, we also evaluated the statistical power for the 496 included cases, and the results showed that the computed statistical power was 0.833.

Patients with SLE were recruited from the Department of Rheumatology and Immunology at Anhui Provincial Hospital, The First Affiliated Hospital of Anhui Medical University. The diagnosis of SLE was established by the presence of four or more 1997 revised American College of Rheumatology (ACR) classification criteria [27]. Patients with viral infections and any history of cancer, pregnancy, and recurrent spontaneous abortions were excluded based on reviews of their appropriate history. The normal controls consisted of age, gender, and ethnicity-matched healthy individuals who belonged to the same geographical area as that of cases; normal controls were excluded if they had a family history of SLE or any other autoimmune disease and history of any chronic or lifestyle diseases like depression, hypothyroidism, hypertension, diabetes mellitus, and tuberculosis (TB). Demographics, clinical features, and related laboratory results were obtained from hospital medical records and then reviewed by experienced physicians.

The Ethical Committee of Anhui Medical University (Hefei, Anhui, China) approved this study. All the study subjects provided informed consent to participate in this study.

All studies on humans described in the present manuscript were carried out with the approval of the responsible ethics committee and in accordance with the national law and the Declaration of Helsinki 1975 (in its current, revised form).

### 2.2. MTN Pathway Gene's SNP Selection and Genotyping

Ensembl Gene Browser 37 (GRCh37) (http://grch37.ensembl.org/index.html) (Ensembl Archive Release 90) was implemented to acquire the genetic and location information of *MTNR1a*, *MTNR1b*, and *AANAT* genes [28], and linkage pedigree file (PED) and marker information file were downloaded. Then, the downloaded files were used to select the tag SNPs in Haploview 4.2 software (Broad Institute, Cambridge, MA, USA), with a MAF above 0.05 in Chinese Han population (CHB) of Beijing, linkage disequilibrium (LD) with an *r*^2^ threshold of 0.8. A total of 46 tag SNPs (23 *MTNR1a* tag SNPs, 13 *MTNR1b* tag SNPs, and 10 *AANAT* tag SNPs) were captured for further evaluation. The function prediction for 46 tag SNPs was assessed by the online bioinformatics tools (https://snpinfo.niehs.nih.gov/snpinfo/snpfunc.html) [29]; the basic information of these tag SNPs is shown in Supplementary [Supplementary-material supplementary-material-1]. In addition, the published literatures about the MTN pathway gene's polymorphisms were also carefully reviewed. Based on one or more of the following criteria: assumed functionality (located in the regulatory regions, for example, 3′-untranslated regions (UTR), 5′-UTR, or amino acid change), *r*^2^ ≥ 0.80 and MAF ≥ 5%, as well as previous studies reported SNPs), the five tag SNPs (rs10030173, rs2119882, rs2165667, rs4861722, and rs6847693) in *MTNR1a* gene, three tag SNPs (rs1562444, rs10830962, and rs3781637) in *MTNR1b* gene, and three tag SNPs (rs8150, rs3760138, and rs12942767) in *AANAT* gene were finally chosen for further genotyping.

Genomic DNA was extracted from peripheral venous blood of patients and healthy controls using a QIAGEN kit (QIAGEN, Hilden, Germany) based on the manufacturer's instructions, and quantification and concentration of DNA was determined using NanoDrop 2000 Spectrophotometer (Thermo Fisher Scientific, USA). Qualified sample requirements were shown as follows: concentration greater than 50 ng/*μ*l, total amount greater than 600 ng, and no obvious degradation.

Genotyping was performed in using improved multiple ligase detection reaction (iMLDR), with technical support from the Center for Genetic & Genomic Analysis, Genesky Biotechnologies Inc., Shanghai. A multiplex PCR-ligase detection reaction method was used in the iMLDR. For each SNP, the alleles were distinguished by different fluorescent labels of allele-specific oligonucleotide probe pairs. Different SNPs were further distinguished by different extended lengths at the 3′ end. Two negative controls were set: one with double-distilled water as template and the other with DNA sample without primers while keeping all other conditions the same in one plate. Duplicate tests were designed, and the results were consistent. A random sample accounting for ~3% of the total DNA samples was directly sequenced using BigDye Terminator version 3.1 and an ABI3730XL automated sequencer (Applied Biosystems) to confirm the results of iMLDR.

### 2.3. Plasma MTN Determination

Blood samples were collected from 5 ml of whole blood of all study subjects and then stored at -80°C until assayed. Plasma MTN concentration was determined by enzyme-linked immunosorbent assay (ELISA) kits (the lower detection limit was 0.1 pg/ml) which were purchased from Anhui Xinle Biotechnology Co. Ltd.; the results of MTN were expressed as picograms per milliliter. The interassay and intraassay variation coefficients of the ELISA kit of our study were 6.9% and 7.6%, respectively.

### 2.4. Statistical Analysis

The allelic and genotypic association analyses between SLE patients and healthy controls were performed in using the Chi-square or Fisher's exact test. Logistic regression analyses were utilized to calculate odds ratios (ORs), and 95% confidence intervals (CIs) for the association between genotype and SLE susceptibility, additive, dominant, recessive, and allelic models were also considered. A nonparametric test was used to compare the difference in plasma MTN levels among patients with different genotypes. Statistical analysis was implemented with the use of the SPSS (IBM Corp. Released 2015. IBM SPSS Statistics for Windows, Version 23.0. Armonk, NY: IBM Corp.).

Hardy–Weinberg equilibrium (HWE) among controls was assessed by comparing the observed-to-expected genotype frequencies using the Chi-square test. Online software SHEsis was used for haplotype analyses of each MTN pathway genes; all the haplotypes with a frequency < 0.03 were ignored in the analysis [30]. All results with a two-tailed *P* < 0.05 were considered to be statistically significant. The Bonferroni correction was used for multiple testing.

## 3. Results

### 3.1. Characteristics of the Study Population

The present study recruited 988 subjects, with 495 SLE patients and 493 healthy controls. In SLE patients, there were 57 males and 438 females with a median age of 37.00 (28.00, 46.00) years and the median disease duration was 4.10 (range from 1.05 to 9.06) years, while there were 55 males and 434 females with the median age of 38.00 (30.00, 47.00) in healthy controls. No significant differences in gender and age distribution were observed between SLE patients and normal controls **(**Table 1**)**. The major clinical manifestations of SLE were immunological abnormality (73.1%), hematological abnormality (68.3%), arthritis (49.5%), malar rash (45.3%), photosensitivity (39.0%), and renal abnormality (37.2%). In control groups, the presence of observed genotype frequencies of all included tag SNPs was distributed in compliance with the HWE (all *P* > 0.05).

### 3.2. Association of MTNR1a/b and AANAT Gene's Polymorphisms with Susceptibility to SLE

There were no significant differences in allele and genotype distribution of 8 tag SNPs in *MTNR1a/b* genes between SLE and healthy controls (all *P* > 0.05) (Table 2). However, when analyzing the allele and genotype frequency of 3 tag SNPs in *AANAT* genes, the results showed that two SNPs of rs8150 and rs3760138 were associated with the risk of SLE susceptibility, where CC carriers of rs8150 had a lower risk as compared to GG (OR = 0.537, 95% CI: 0.361, 0.799) (*P* = 0.002), whereas GG carrier of rs3760138 had an increased risk as compared to TT (OR = 1.823, 95% CI: 1.154, 2.880) (*P* = 0.010), but we did not observe other positive findings regarding the SNPs of rs12942767 (Table 3).

### 3.3. Association of MTNR1a/b and AANAT Gene's Polymorphisms with Clinical Features in Patients with SLE

Case-only analysis was conducted to further explore the genetic association of *MTNR1a/b* and *AANAT* gene's polymorphisms with specific clinical features of SLE. In *MTNR1a/b* genes, a significantly increased AA genotype frequency of rs2165667 (*MTNR1a*) and A allele frequency of rs1562444 (*MTNR1b*) were found in patients with arthritis than those without (both *P* = 0.024). In addition, the frequency of CC/CG genotype in rs10830962 (*MTNR1b*) was significantly lower in patients with malar rash than in those without (*P* = 0.018). In terms of the genetic association of *AANAT* gene with clinical features of SLE, there was a higher GG/GT/TT genotype distribution of rs3760138 in patients with positive immunological abnormality than those with negative (*P* = 0.024); the C/G allele and CC/CG/GG genotype frequency of rs8150 appeared to have a significantly increased risk in patients with positive hematological abnormality compared with those with negative (*P* = 0.039, *P* = 0.010, respectively). Nevertheless, no other positive findings were revealed regarding the *MTNR1a/b* and *AANAT* gene's polymorphisms with SLE clinical features (Table 4).

### 3.4. Association of Plasma MTN Concentrations with Genotypes in Patients with SLE

The results indicated that, in patients with SLE, there was a significant difference of MTN level among genotype of AA, AG, and GG in rs1562444 (*MTNR1b*) (*P* = 0.001), which GG genotype showed an elevated MTN concentration than in AA and AG genotype (20.57 pg/ml *vs* 14.08 pg/ml *vs* 10.36 pg/ml). However, no significant differences of plasma MTN concentrations were observed in other SNPs between patients (Table 5).

### 3.5. Haplotype Analyses

The haplotype of tag SNPs in *MTNR1a/b* and *AANAT* genes was constructed by using SHEsis software. Haplotype analyses implied that haplotype CCTAT (*MTNR1a*), CTAGT (*MTNR1a*), and GGG (*AANAT*) were significantly associated with the increased risk in SLE susceptibility, but TCTAT (*MTNR1a*) and CTG (*AANAT*) appeared to be a protective haplotype (all *P* < 0.05). However, no positive findings of other haplotypes were observed (Tables 6–8).

## 4. Discussion

The neuroendocrine-immune system is regarded as a fundamental network supporting the health state that could play an important role in the development of autoimmune disorders [31, 32]. MTN, as one of the pineal gland-driven hormones, secreted in a circadian rhythm and regulated by photoperiod with the highest peak at midnight and lowest level after sunrise, acting mainly as a regulator for sleeping rhythm [33–36]. In recent years, the bidirectional associations between the pineal gland and the immune system have been suggested to depend on the immune-modulating effect of MTN and the pineal regulation by different lymphokines [37]. MTN might have a direct effect on immune-competent cells, thus, fulfilling critical roles on the development and progression of autoimmune diseases [38–40].

Our previous study has evaluated the level of MTN in patients with SLE as compared to healthy controls. Although there was no significant difference in MTN concentration between those two groups, we observed a slightly lower level of MTN in SLE patients than in healthy controls; in addition, an inverse correlation of MTN concentration with IgM was also revealed [41]. Similarly, Robeva et al. revealed that there was a decreased daily MTN level in women with SLE and found an inverse relationship between daily MTN concentrations and disease activity [19]. In the subarctic region, the study has demonstrated the presence of seasonal variations in daily MTN level, where the increased level of MTN was discovered in December than that in June [20]. These evidences suggest that the change of MTN concentration may be involved in the pathogenesis of SLE.

*MTNR1a/b* and *AANAT* play an important role during the pathway of MTN from biosynthesis to its functioning; the former *MTNR1a/b* genes are largely responsible for mediating the downstream effects of MTN, while the latter *AANAT* gene is the major enzyme in MTN synthesis. Several previous studies have investigated the underlying role of MTN pathway genes in a number of human diseases. In SLE, primary study, performed by Tanev et al., has demonstrated no significant differences in allelic and genotype distribution of *MTNR1b* gene (rs1562444, rs10830962, and rs10830963) polymorphisms between 109 patients with SLE and 101 controls, yet, in SLE patients, C/C genotype of rs10830963 in *MTNR1b* gene was related to increased prevalence of leucopenia compared to C/G and G/G genotype; the rs10830963 G/G carriers had a lower number of lupus criteria than in those with C/C genotype [25]. The *MTNR1b* rs10830962 and rs10830963 polymorphisms have been predominantly investigated in the context of metabolic disorders, of which rs10830962 and rs10830963 G alleles were reported to associate with reduced insulin secretion, increased fasting plasma glucose concentrations, and increased risk for diabetes in different populations [42–44]. In autoimmune diseases of multiple sclerosis (MS), there were no significant allelic associations of SNPs rs4753426 and rs10830963 in *MTNR1b* gene with susceptibility to MS, but the rs10830963-rs4753426 G-T haplotype associated with the risk of MS in the progressive MS group [23]. Deming et al. analyzed the MTN pathway gene's polymorphisms in human breast cancer patients, and they supported that AA genotype of *MTNR1b* rs10765576 was associated with a decreased risk of breast cancer, the GG genotype in premenopausal women correlated with an increased risk for breast cancer, and however, in postmenopausal women, the GG genotype were related with a decreased risk of breast cancer; they did not observe any significant breast cancer associations for variants in the *AANAT* gene [24]. In patients with major depression, the two SNPs of *AANAT* (rs3760138 and rs4238989) were reported to be associated with an increased contribution to major depression [26].

In the present study, the tag SNPs of rs8150 and rs3760138 in *AANAT* gene were associated with genetic susceptibility to SLE, but no genetic association regarding the other nine tag SNPs with SLE susceptibility was found. Case-only analysis indicated that AA genotype frequency in rs2165667 (*MTNR1a*) AA genotype and rs1562444 (*MTNR1b*) A/G allele frequency were at increased risk for arthritis and rs10830962 (*MTNR1b*) CC/CG genotype was at decreased risk for malar rash. In *AANAT* gene, rs3760138 GG/GT/TT genotype associated with positive immunological abnormality than those with negative, and rs8150 CC/CG/GG genotype and its C/G allele appeared to have an increased risk for hematological abnormality. Moreover, we also found that there was a significant difference of MTN concentration samong the genotype of AA, AG, and GG in rs1562444 (*MTNR1b*), where the GG genotype showed an elevated MTN concentration than in AA and AG genotype. We might hypothesize that the rs1562444 variant polymorphisms lead to the aberrant expression of MTN in patients with SLE. Later, the haplotype of *MTNR1a/b* and *AANAT* was identified; *MTNR1a* gene haplotype of CCTAT and CTAGT, and *AANAT* gene haplotype of GGG showed an increased risk in SLE susceptibility, but haplotype of TCTAT (*MTNR1a*) and CTG (*AANAT*) appeared to have a protective role.

The present study investigated the genetic association of SNPs in MTN pathway genes (*MTNR1a/b* and *AANAT*) with SLE susceptibility. However, there are some limitations in our study. First, the current study might be due to inherent selection biases such as a relative small sample size, the limited number of variables accounted, and the lack of information regarding body mass index (BMI) in healthy controls. Second, the potential confounding factors, such as type of treatments and concomitant infections, may have an effect on the level of MTN. Furthermore, although our study represented significant genetic variations regarding *AANAT* gene in Chinese SLE patients, the detailed mechanism about potential effect of the *AANAT* gene variation on SLE is scarce.

In conclusion, our study demonstrated that, in the Chinese population, the genetic polymorphism of MTN pathway genes associated with the susceptibility to SLE, as well as with specific clinical manifestations, suggesting that the MTN pathway genes might be involved in the pathogenesis and development of SLE. However, further large sample size studies in other population are needed to further reveal the significance of MTN pathway gene's polymorphisms in SLE. In addition, related mechanism researches are necessary to better understand the function of the MTN pathway gene SNP in different immune cell types and to evaluate its correlation with clinical features.

## Figures and Tables

**Table 1:** Demographic characteristics and clinical features of patients with SLE and control subjects.

Parameters	Patients with SLE (*n* = 495)	Healthy controls (*n* = 493)
Demographic characteristics		
Age (year)	37.00 (28.00, 46.00)^∗∗^	38.00 (30.00, 47.00) ^∗∗^
Female, *n* (%)	438 (88.5)	434 (88.0)
Male, *n* (%)	58 (11.5)	59 (12.0)
Disease duration (year)	4.10 (1.05, 9.06) ^∗∗^	NA
BMI (kg/m^2^)	21.48 (19.72, 23.43) ^∗∗^	NA
SLEDAI	11.40 ± 9.07^∗^	NA
Disease manifestations		NA
Malar rash, *n* (%)	224 (45.3)	NA
Discoid rash, *n* (%)	94 (19.0)	NA
Photosensitivity, *n* (%)	193 (39.0)	NA
Oral ulcers, *n* (%)	119 (24.0)	NA
Arthritis, *n* (%)	245 (49.5)	NA
Serositis, *n* (%)	45 (9.1)	NA
Renal abnormality, *n* (%)	184 (37.2)	NA
Neurological abnormality, *n* (%)	21 (4.2)	NA
Hematological abnormality, *n* (%)	338 (68.3)	NA
Immunological abnormality, *n* (%)	362 (73.1)	NA

BMI: Body mass index; *n*: number; SLE: systemic lupus erythematosus; SLEDAI: systemic lupus erythematosus disease activity index. ^∗^Data with normal distribution were described as mean and standard deviation (SD). ^∗∗^Data with skewed distribution were described as median and interquartile range (IQR).

**Table 2:** Genotype frequencies of *MTNR1a/b* SNPs in SLE patients and healthy controls.

SNPs	Analyzed model		SLE	Control	OR (95% CI)	*P* value^∗^
rs10030173 (*MTNR1a*)	Genotypes	CC	107	93	1.308 (0.916, 1.867)	0.139
		CT	242	234	1.176 (0.883, 1.565)	0.267
		TT	146	166	1.000	
	Additive model	CC	107	93	1.308 (0.916, 1.867)	0.139
		TT	146	166	1.000	

rs2119882 (*MTNR1a*)	Genotypes	CC	71	78	0.792 (0.542, 1.156)	0.226
		CT	217	235	0.803 (0.612, 1.054)	0.114
		TT	207	180	1.000	
	Additive model	CC	71	78	0.792 (0.542, 1.156)	0.226
		TT	207	180	1.000	

rs2165667 (*MTNR1a*)	Genotypes	AA	230	200	1.267 (0.849, 1.890)	0.246
		AT	206	228	0.995 (0.667, 1.484)	0.982
		TT	59	65	1.000	
	Additive model	AA	230	200	1.267 (0.849, 1.890)	0.246
		TT	59	65	1.000	

rs4861722 (*MTNR1a*)	Genotypes	AA	25	28	0.978 (0.548, 1.743)	0.939
		GA	165	189	1.243 (0.707, 2.183)	0.450
		GG	304	274	1.000	
	Additive model	AA	25	28	0.978 (0.548, 1.743)	0.939
		GG	304	274	1.000	

rs6847693 (*MTNR1a*)	Genotypes	CC	182	167	0.868 (0.658, 1.145)	0.318
		CT	229	242	0.918 (0.635, 1.326)	0.647
		TT	84	84	1.000	
	Additive model	CC	182	167	0.868 (0.658, 1.145)	0.318
		TT	84	84	1.000	

rs1562444 (*MTNR1b*)	Genotypes	AA	254	262	1.039 (0.659, 1.636)	0.870
		GA	199	186	1.146 (0.720, 1.826)	0.565
		GG	42	45	1.000	
	Additive model	AA	254	262	1.039 (0.659, 1.636)	0.870
		GG	42	45	1.000	

rs10830962 (*MTNR1b*)	Genotypes	CC	176	145	1.349 (0.951, 1.913)	0.093
		CG	220	236	1.036 (0.746, 1.438)	0.834
		GG	99	110	1.000	
	Additive model	CC	176	145	1.349 (0.951, 1.913)	0.093
		GG	99	110	1.000	

rs3781637 (*MTNR1b*)	Genotypes	CC	11	11	1.009 (0.420, 2.424)	0.984
		CT	111	110	1.003 (0.429, 2.341)	0.995
		TT	373	372	1.000	
	Additive model	CC	11	11	1.009 (0.420, 2.424)	0.984
		TT	373	372	1.000	

SLE: systemic lupus erythematosus; SNPs: single-nucleotide polymorphisms; OR: odds ratio; *MTNR1a*: *melatonin receptor 1a*; *MTNR1b*: *melatonin receptor 1b.*^∗^The *P* values are not corrected for multiple testings, Bonferroni corrected *P* = 0.0167.

**Table 3:** Genotype frequencies of *AANAT* SNPs in SLE patients and healthy controls.

SNPs	Analyzed model		SLE	Control	OR (95% CI)	*P* value^∗^
rs8150	Genotypes	CC	176	202	0.537 (0.361, 0.799)	**0.002**
		GC	232	236	1.128 (0.860, 1.480)	0.384
		GG	86	53	1.000	
	Additive model	CC	176	202	0.537 (0.361, 0.799)	**0.002**
		GG	86	53	1.000	

rs3760138	Genotypes	GG	271	223	1.823 (1.154, 2.880)	**0.010**
		GT	188	216	1.306 (0.820, 2.078)	0.261
		TT	36	54	1.000	
	Additive model	GG	271	223	1.823 (1.154, 2.880)	**0.010**
		TT	36	54	1.000	

rs12942767	Genotypes	GG	450	447	—	1.000
		GA	44	46	—	1.000
		AA	1	0	1.000	
	Additive model	GG	450	447	—	1.000
		AA	1	0	1.000	

SLE: systemic lupus erythematosus; SNPs: single-nucleotide polymorphisms; OR: odds ratio; *AANAT*: *arylalkylamine N-acetyltransferase*. ^∗^The *P* values are not corrected for multiple testings, Bonferroni corrected *P* = 0.0167.

**Table 4:** The positive findings on association of clinical characteristics with genotype and allele frequencies in *MTNR1a/b* and *AANAT* genes.

Gene (SNPs)	Allele (M/m)	Clinical features	Group	Genotypes (*n*)			*P* value	Alleles (*n*)		*P* value
				MM	Mm	mm		*M*	*m*	
rs2165667 (*MTNR1a*)	A/T	Arthritis	Positive	125	87	33	**0.024**	337	153	0.318
			Negative	105	119	26		329	171	

rs10830962 (*MTNR1b*)	C/G	Malar rash	Positive	77	90	57	**0.018**	244	204	0.055
			Negative	99	130	42		328	214	

rs1562444 (*MTNR1b*)	A/G	Arthritis	Positive	136	94	15	0.072	366	124	**0.024**
			Negative	118	105	27		341	159	

rs3760138 (*AANAT*)	G/T	Immunological abnormality	Positive	198	131	33	**0.024**	527	197	0.264
			Negative	73	57	3		203	63	

rs8150 (*AANAT*)	C/G	Hematological abnormality	Positive	131	155	51	**0.039**	417	257	**0.010**
			Negative	45	77	35		167	147	

SNPs: single-nucleotide polymorphisms; *MTNR1a*: *melatonin receptor 1a*; *MTNR1b*: *melatonin receptor 1b*; *AANAT*: *arylalkylamine N-acetyltransferase.*

**Table 5:** Association of plasma MTN levels with genotype in *MTNR1a/b* and *AANAT.*

SNPs	Genotypes	Number	Plasma MTN levels (pg/ml)	*P* value
			*M* (*P*_25_, *P*_75_)	
rs10030173 (*MTNR1a*)	CC	19	14.22 (9.23, 20.57)	0.946
	CT	45	13.45 (9.91, 19.06)	
	TT	19	12.39 (10.27, 18.69)	

rs2119882 (*MTNR1a*)	CC	9	12.19 (9.54, 25.08)	0.312
	CT	44	13.79 (10.34, 20.00)	
	TT	30	10.82 (9.16, 16.33)	

rs2165667 (*MTNR1a*)	AA	32	10.69 (8.41, 16.12)	0.079
	AT	44	13.24 (10.39, 19.91)	
	TT	7	19.38 (10.49, 28.21)	

rs4861722 (*MTNR1a*)	AA	6	15.47 (8.24, 34.58)	0.640
	GA	30	11.74 (10.19, 16.35)	
	GG	47	13.71 (9.91, 21.46)	

rs6847693 (*MTNR1a*)	CC	25	10.62 (9.08, 16.41)	0.333
	CT	47	13.45 (10.36, 20.20)	
	TT	11	18.37 (8.59, 21.95)	
	CT	1	11.07	

rs1562444 (*MTNR1b*)	AA	41	14.08 (10.63, 20.08)	**0.001**
	AG	35	10.36 (8.94, 13.86)	
	GG	7	20.57 (18.37, 28.21)	

rs10830962 (*MTNR1b*)	CC	25	13.71 (10.32, 22.10)	0308
	CG	49	11.58 (9.80, 18.30)	
	GG	9	15.57 (10.05, 33.40)	

rs3781637 (*MTNR1b*)	CC	3	18.37 (13.71, 28.21)	0.392
	CT	16	13.12 (8.57, 20.27)	
	TT	64	12.55 (10.25, 17.83)	

rs8150 (*AANAT*)	CC	36	13.68 (10.28, 23.20)	0.518
	GC	34	11.88 (9.62, 18.45)	
	GG	13	13.04 (10.44, 14.82)	

rs3760138 (*AANAT*)	GG	45	12.19 (9.78, 17.09)	0.802
	GT	31	13.45 (10.05, 21.95)	
	TT	7	14.27 (12.01, 21.46)	

rs12942767 (*AANAT*)	GA	5	13.03 (9.88, 16.83)	0.878
	GG	78	13.07 (9.92, 19.59)	

SNPs: single-nucleotide polymorphisms; *M*: median; MTN: melatonin; *MTNR1a*: *melatonin receptor 1a*; *MTNR1b*: *melatonin receptor 1b*; *AANAT*: *arylalkylamine N-acetyltransferase.*

**Table 6:** Haplotype analysis of five SNPs in *MTNR1a* gene in SLE patients and healthy controls.

Haplotype	SLE (*n* (%))	Controls (*n* (%))	*χ* ^2^	*P*	OR (95% CI)
rs10030173, rs2119882, rs2165667, rs4861722, rs6847693					
CCTAT	44.05 (0.045)	27.21 (0.028)	4.135	**0.042031**	1.650 (1.014, 2.686)
CTAGC	341.42 (0.346)	331.12 (0.337)	0.241	0.623479	1.049 (0.866, 1.270)
CTAGT	38.30 (0.039)	19.92 (0.020)	5.983	**0.014466**	1.962 (1.133, 3.400)
TCTAT	135.01 (0.137)	180.70 (0.184)	8.071	**0.004512**	0.702 (0.549, 0.897)
TCTGT	117.73 (0.119)	118.09 (0.120)	0.001	0.974785	0.996 (0.757, 1.309)
TTAGC	217.53 (0.220)	216.35 (0.220)	0.003	0.954914	1.006 (0.811, 1.249)

SLE: systemic lupus erythematosus; SNPs: single-nucleotide polymorphisms; *MTNR1a*: *melatonin receptor 1a*; OR: odds ratio. Total *χ*^2^ = 16.556, *P* = 0.005. All the haplotypes with a frequency < 0.03 were ignored in the analysis.

**Table 7:** Haplotype analysis of three SNPs in *MTNR1b* gene in SLE patients and healthy controls.

Haplotype	SLE (*n* (%))	Control (*n* (%))	*χ* ^2^	*P*	OR (95% CI)
rs1562444, rs10830962, rs3781637					
CAT	296.17 (0.299)	260.80 (0.265)	2.573	0.108701	1.175 (0.965, 1.430)
CGC	130.72 (0.132)	127.39 (0.129)	0.014	0.906006	1.016 (0.782, 1.320)
CGT	144.94 (0.146)	137.77 (0.140)	0.123	0.725622	1.046 (0.813, 1.346)
GAT	408.55 (0.413)	442.60 (0.450)	3.208	0.073303	0.849 (0.710, 1.016)

SLE: systemic lupus erythematosus; SNPs: single-nucleotide polymorphisms; *MTNR1b*: *melatonin receptor 1b*; OR: odds ratio. Total *χ*^2^ = 3.762, *P* = 0.288. All the haplotypes with a frequency < 0.03 were ignored in the analysis.

**Table 8:** Haplotype analysis of three SNPs in AANAT gene in SLE patients and healthy controls.

Haplotype	SLE (*n* (%))	Control (*n* (%))	*χ* ^2^	*P*	OR (95% CI)
rs8150, rs3760138, rs12942767					
CGG	339.49 (0.344)	324.11 (0.329)	0.559	0.454842	1.074 (0.891, 1.295)
CTG	244.50 (0.247)	315.27 (0.320)	12.475	**0.000415**	0.701 (0.576, 0.854)
GGA	43.12 (0.044)	44.37 (0.045)	0.018	0.894335	0.971 (0.633, 1.492)
GGG	347.39 (0.352)	291.50 (0.296)	7.336	**0.006775**	1.299 (1.075, 1.571)

SLE: systemic lupus erythematosus; SNPs: single-nucleotide polymorphisms; *AANAT*: *arylalkylamine N-acetyltransferase*; OR: odds ratio. Total *χ*^2^ = 14.211, *P* = 0.003. All the haplotypes with a frequency < 0.03 were ignored in the analysis.

## Data Availability

The data that support the findings of this study are available from the corresponding author upon reasonable request.

## Supplementary Materials

Supplementary Materials Supplementary Table 1: characteristics of the 46 Tag SNPs.
