# Supplementary material for: Association of Melatonin Pathway Gene's Single-Nucleotide Polymorphisms with Systemic Lupus Erythematosus in a Chinese Population
Source: J Immunol Res. 2019 Nov 13;2019:2397698. doi: 10.1155/2019/2397698 (PMC6877953; doi:10.1155/2019/2397698)
Supplement: Supplementary Materials — Supplementary Table 1: characteristics of the 46 Tag SNPs. [file 2397698.f1.docx]

**Supplementary Table 1** Characteristics of the 46 Tag SNPs

| rs | Chr | Position | Allele | TFBS | Splicing  (site) | Splicing  (ESE or ESS) | Splicing  (abolish domain) | miRNA  (miRanda) | miRNA  (Sanger) | nsSNP | Stop Codon | Polyphen | SNPs3D  (svm profile) | SNPs3D  (svm structure) | RegPotential | Conservation | Nearby Gene | Distance (bp) | Allele | CHB |
| --- | --- | --- | --- | --- | --- | --- | --- | --- | --- | --- | --- | --- | --- | --- | --- | --- | --- | --- | --- | --- |
| rs10005645 | 4 | 187476900 | C/T | -- | -- | -- | -- | -- | -- | -- | -- | -- | -- | -- | NA | 0.023 | F11\|\|LOC644042 | -29071\|\|-9903 | T | 0.395 |
| rs10020303 | 4 | 187472992 | A/G | -- | -- | -- | -- | -- | -- | -- | -- | -- | -- | -- | NA | 0 | F11\|\|LOC644042 | -25163\|\|-13811 | A | 0.765 |
| rs10029715 | 4 | 187459594 | C/T | -- | -- | -- | -- | -- | -- | -- | -- | -- | -- | -- | NA | 0.017 | F11\|\|LOC644042 | -11765\|\|-27209 | T | 0.792 |
| rs10030173 | 4 | 187730809 | C/T | -- | -- | -- | -- | -- | -- | -- | -- | -- | -- | -- | NA | 0.002 | MTNR1A\|\|FAT | -17278\|\|-15122 | T | 0.571 |
| rs1008728 | 4 | 187463667 | C/T | -- | -- | -- | -- | -- | -- | -- | -- | -- | -- | -- | NA | 0 | F11\|\|LOC644042 | -15838\|\|-23136 | T | 0.756 |
| rs11132387 | 4 | 187455717 | A/G | -- | -- | -- | -- | -- | -- | -- | -- | -- | -- | -- | NA | 0 | F11\|\|LOC644042 | -7888\|\|-31086 | G | 0.274 |
| rs12331264 | 4 | 187470832 | C/T | -- | -- | -- | -- | -- | -- | -- | -- | -- | -- | -- | NA | 0.011 | F11\|\|LOC644042 | -23003\|\|-15971 | T | 0.669 |
| rs12500826 | 4 | 187464445 | C/T | -- | -- | -- | -- | -- | -- | -- | -- | -- | -- | -- | NA | 0 | F11\|\|LOC644042 | -16616\|\|-22358 | C | 0.872 |
| rs12507040 | 4 | 187454367 | A/G | -- | -- | -- | -- | -- | -- | -- | -- | -- | -- | -- | NA | 0.006 | F11\|\|LOC644042 | -6538\|\|-32436 | G | 0.845 |
| rs13133050 | 4 | 187467731 | A/C | -- | -- | -- | -- | -- | -- | -- | -- | -- | -- | -- | NA | 0 | F11\|\|LOC644042 | -19902\|\|-19072 | C | 0.839 |
| rs13145616 | 4 | 187452010 | A/C | -- | -- | -- | -- | -- | -- | -- | -- | -- | -- | -- | NA | 0 | F11\|\|LOC644042 | -4181\|\|-34793 | C | 0.964 |
| rs2119882 | 4 | 187713899 | C/T | Y | -- | -- | -- | -- | -- | -- | -- | -- | -- | -- | NA | 0.001 | MTNR1A\|\|FAT | -368\|\|-32032 | T | 0.5 |
| rs2165667 | 4 | 187698623 | T/A | -- | -- | -- | -- | -- | -- | -- | -- | -- | -- | -- | NA | 0 | MTNR1A | 6820\|\|14908 | T | 0.422 |
| rs4861722 | 4 | 187726377 | A/G | -- | -- | -- | -- | -- | -- | -- | -- | -- | -- | -- | NA | 0.011 | MTNR1A\|\|FAT | -12846\|\|-19554 | G | 0.738 |
| rs6552971 | 4 | 187475382 | A/G | -- | -- | -- | -- | -- | -- | -- | -- | -- | -- | -- | NA | 0 | F11\|\|LOC644042 | -27553\|\|-11421 | G | 0.851 |
| rs6552972 | 4 | 187475398 | C/T | -- | -- | -- | -- | -- | -- | -- | -- | -- | -- | -- | NA | 0.002 | F11\|\|LOC644042 | -27569\|\|-11405 | T | 0.923 |
| rs6839415 | 4 | 187474299 | C/T | -- | -- | -- | -- | -- | -- | -- | -- | -- | -- | -- | NA | 0.02 | F11\|\|LOC644042 | -26470\|\|-12504 | T | 0.845 |
| rs6847693 | 4 | 187698638 | C/T | -- | -- | -- | -- | -- | -- | -- | -- | -- | -- | -- | NA | 0 | MTNR1A | 6835\|\|14893 | T | 0.452 |
| rs6848311 | 4 | 187455812 | G/T | -- | -- | -- | -- | -- | -- | -- | -- | -- | -- | -- | NA | 0.035 | F11\|\|LOC644042 | -7983\|\|-30991 | G | 0.815 |
| rs6854650 | 4 | 187472947 | C/T | -- | -- | -- | -- | -- | -- | -- | -- | -- | -- | -- | NA | 0 | F11\|\|LOC644042 | -25118\|\|-13856 | C | 0.463 |
| rs7687352 | 4 | 187476741 | A/G | -- | -- | -- | -- | -- | -- | -- | -- | -- | -- | -- | NA | 0.002 | F11\|\|LOC644042 | -28912\|\|-10062 | A | 0.671 |
| rs7700014 | 4 | 187478491 | C/T | -- | -- | -- | -- | -- | -- | -- | -- | -- | -- | -- | NA | 0.001 | F11\|\|LOC644042 | -30662\|\|-8312 | T | 0.687 |
| rs9993749 | 4 | 187476843 | G/T | -- | -- | -- | -- | -- | -- | -- | -- | -- | -- | -- | NA | 0.048 | F11\|\|LOC644042 | -29014\|\|-9960 | G | 0.53 |
| rs10830962 | 11 | 92338075 | C/G | Y | -- | -- | -- | -- | -- | -- | -- | -- | -- | -- | 0 | 0 | LOC100128354\|\|MTNR1B | -27591\|\|-4362 | G | 0.411 |
| rs10831027 | 11 | 92708597 | C/T | -- | -- | -- | -- | -- | -- | -- | -- | -- | -- | -- | NA | 0.002 | CCDC67 | 5066\|\|102687 | T | 0.268 |
| rs12419719 | 11 | 92710328 | A/G | -- | -- | -- | -- | -- | -- | -- | -- | -- | -- | -- | 0 | 0 | CCDC67 | 6797\|\|100956 | A | 0.845 |
| rs1562444 | 11 | 92355497 | A/G | -- | -- | -- | -- | -- | -- | -- | -- | -- | -- | -- | 0 | 0 | MTNR1B | 13060\|\|99 | A | 0.679 |
| rs3019210 | 11 | 92699092 | T/G | Y | -- | -- | -- | -- | -- | -- | -- | -- | -- | -- | 0 | 0.004 | LOC100128230\|\|CCDC67 | -128171\|\|-4439 | G | 0.381 |
| rs3019218 | 11 | 92716408 | C/G | -- | -- | -- | -- | -- | -- | -- | -- | -- | -- | -- | 0.002001 | 0 | CCDC67 | 12877\|\|94876 | G | 0.488 |
| rs3019219 | 11 | 92718766 | A/T | -- | -- | -- | -- | -- | -- | -- | -- | -- | -- | -- | NA | 0.876 | CCDC67 | 15235\|\|92518 | T | 0.57 |
| rs3019221 | 11 | 92719348 | C/G | -- | -- | -- | -- | -- | -- | -- | -- | -- | -- | -- | NA | 0.003 | CCDC67 | 15817\|\|91936 | G | 0.946 |
| rs3020061 | 11 | 92702848 | T/C | Y | -- | -- | -- | -- | -- | -- | -- | -- | -- | -- | 0.187942 | 0 | LOC100128230\|\|CCDC67 | -131927\|\|-683 | C | 0.911 |
| rs3020066 | 11 | 92698133 | G/C | -- | -- | -- | -- | -- | -- | -- | -- | -- | -- | -- | NA | 0 | LOC100128230\|\|CCDC67 | -127212\|\|-5398 | C | 0.822 |
| rs3781637 | 11 | 92353418 | T/C | -- | -- | -- | -- | -- | -- | -- | -- | -- | -- | -- | 0 | 0.007 | MTNR1B | 10981\|\|2178 | T | 0.9 |
| rs4322357 | 11 | 92712491 | A/C | -- | -- | -- | -- | -- | -- | -- | -- | -- | -- | -- | 0.078514 | 0 | CCDC67 | 8960\|\|98793 | C | 0.869 |
| rs7949194 | 11 | 92707406 | A/G | -- | -- | -- | -- | -- | -- | -- | -- | -- | -- | -- | 0 | 0.002 | CCDC67 | 3875\|\|103878 | A | 0.911 |
| rs11654023 | 17 | 74467863 | A/C | -- | -- | -- | -- | -- | -- | -- | -- | -- | -- | -- | 0 | 0.001 | TIMP2\|\|LOC100133045 | -34796\|\|-11051 | C | 0.744 |
| rs12942767 | 17 | 71980147 | A/G | -- | -- | -- | -- | -- | -- | -- | -- | -- | -- | -- | 0 | 0 | RHBDF2 | 1577\|\|28956 | G | 0.933 |
| rs3760138 | 17 | 71974704 | G/T | Y | -- | -- | -- | -- | -- | -- | -- | -- | -- | -- | 0 | 0.085 | UBE2O\|\|AANAT | -13821\|\|-542 | G | 0.767 |
| rs4789915 | 17 | 74460186 | T/G | -- | -- | -- | -- | -- | -- | -- | -- | -- | -- | -- | 0.138064 | 0 | TIMP2\|\|LOC100133045 | -27119\|\|-18728 | T | 0.929 |
| rs4789916 | 17 | 74460096 | G/A | -- | -- | -- | -- | -- | -- | -- | -- | -- | -- | -- | 0.223876 | 0 | TIMP2\|\|LOC100133045 | -27029\|\|-18818 | G | 0.565 |
| rs4789921 | 17 | 74451214 | T/C | -- | -- | -- | -- | -- | -- | -- | -- | -- | -- | -- | 0 | 0 | TIMP2\|\|LOC100133045 | -18147\|\|-27700 | C | 0.946 |
| rs7208912 | 17 | 74455624 | G/T | -- | -- | -- | -- | -- | -- | -- | -- | -- | -- | -- | NA | 0 | TIMP2\|\|LOC100133045 | -22557\|\|-23290 | G | 0.708 |
| rs7216022 | 17 | 74451104 | C/T | -- | -- | -- | -- | -- | -- | -- | -- | -- | -- | -- | 0 | 0 | TIMP2\|\|LOC100133045 | -18037\|\|-27810 | T | 0.548 |
| rs7221906 | 17 | 74466743 | C/T | -- | -- | -- | -- | -- | -- | -- | -- | -- | -- | -- | NA | 0 | TIMP2\|\|LOC100133045 | -33676\|\|-12171 | C | 0.548 |
| rs8150 | 17 | 71978612 | G/C | -- | -- | Y | -- | Y | -- | -- | -- | -- | -- | -- | 0.09793 | 0.597 | RHBDF2 | 42\|\|30491 | G | 0.393 |

Chr: chromosome; TFBS: transcription factor binding sites; ESE: exonic splicing enhancers; ESS: exonic splicing silencers; nsSNP: nonsynonymous single nucleotide polymorphism; CHB: Chinese Han Beijing.

The function prediction for 46 tag SNPs was assessed by the online bioinformatics tools (<https://snpinfo.niehs.nih.gov/snpinfo/snpfunc.html>)
